# Lamellar ordering, droplet formation and phase inversion in exotic active emulsions

**DOI:** 10.1038/s41598-019-39190-6

**Published:** 2019-02-26

**Authors:** F. Bonelli, L. N. Carenza, G. Gonnella, D. Marenduzzo, E. Orlandini, A. Tiribocchi

**Affiliations:** 1 Dipartimento di Meccanica, Matematica e Management, DMMM, Politecnico di Bari, 70125 Bari, Italy; 2 Dipartimento di Fisica, Universitá degli Srudi di Bari and INFN, Sezione di Bari, Via Amendola 173, 70126 Bari, Italy; 3 SUPA, School of Physics and Astronomy, University of Edinburgh, Edinburgh, EH9 3JZ United Kingdom; 4 Dipartimento di Fisica e Astronomia, Universitá di Padova, 35131 Padova, Italy; 5 Center for Life Nano Science @Sapienza, Istituto Italiano di Tecnologia, Viale Regina Elena, 295, I-00161 Roma, Italy

## Abstract

We study numerically the behaviour of a two-dimensional mixture of a passive isotropic fluid and an active polar gel, in the presence of a surfactant favouring emulsification. Focussing on parameters for which the underlying free energy favours the lamellar phase in the passive limit, we show that the interplay between nonequilibrium and thermodynamic forces creates a range of multifarious exotic emulsions. When the active component is contractile (e.g., an actomyosin solution), moderate activity enhances the efficiency of lamellar ordering, whereas strong activity favours the creation of passive droplets within an active matrix. For extensile activity (occurring, e.g., in microtubule-motor suspensions), instead, we observe an emulsion of spontaneously rotating droplets of different size. By tuning the overall composition, we can create high internal phase emulsions, which undergo sudden phase inversion when activity is switched off. Therefore, we find that activity provides a single control parameter to design composite materials with a strikingly rich range of morphologies.

## Introduction

Active matter has established itself as a topical area of research in physics over the last few years^1,2^. Active systems are internally driven, and continuously take up energy from their surrounding so that they function far from thermal equilibrium. Examples are bacterial swarms^3^, cell extracts^4^, cytoskeletal gels^5,6^, living liquid crystals^7^ and chemically driven phoretic colloids^8–10^. Their inherent non-equilibrium nature causes a range of unexpected behaviours, such as spontaneous flows^11–13^, bacterial turbulence^3,14^ and motility-induced phase separation^15–17^.

While single-component active systems have received a lot of attention, much less is currently known about the behaviour of mixtures, made up by a combination of active and passive components. Mixtures of self-propelled and passive colloidal spheres and rods have been studied via particle-based simulations in refs^18–20^, finding that activity can drive self-assembly or may trigger segregation between the passive and active components. Lyotropic binary mixtures with an active component have also been considered in refs^21–25^ within a continuum model, showing that activity creates self-motile droplets, and may also cause an undulatory instability of the passive-active interface. In general, these composite materials bear great promise as self-assembling new soft materials, as activity allows the system to bypass thermodynamic constraints which would otherwise govern its behaviour, and leads to novel phenomena.

Here we study a theoretical model for a new kind of active material, made up by mixing an isotropic passive fluid with a polar active one, in the presence of a surfactant which favours emulsification. The model is based on a generalization of the Brazovskii free-energy functional^26^ useful to describe complex fluids where the presence of interfaces is favored. We name the resulting composite material an “exotic active emulsion” (see section Methods). There are two potential avenues to realise these systems in the lab. The first is by dispersing sticky bacteria^27^ or self-attractive cytoskeletal gels^5,6^ (the active fluid) in water (the isotropic component), under conditions promoting microphase separation (e.g., depletion forces^5,6,27^ and a suitable surfactant). The second route is through activation of passive emulsions: for instance, it is now possible to encapsulate an active nematic gel within a water-in-oil emulsion^5,6^. This is a striking example of system in which the active network, after being absorbed onto the droplet surface, yields an effectively 2D active nematic, whose physics can be approximately studied by means of 2D simulations similar to those reported here. Additionally, two-dimensional simulations are often adopted to study systems under strong confinement, such as bacterial suspensions^14^, biofilms^28,29^ and eukarytic cells crawling on solid surfaces^30^. We shall comment more on potential experiments at the end of the work.

Active fluids can be either contractile (actomyosin gels^31^) or extensile (bacteria^27^ or microtubule-kinesin^5,6,32^ suspensions), according to the nature of the internal driving force^1,2^. If gravity (which is usually much smaller than other forces at these scales) is neglected, the simplest force field exerted by an active particle is that of a dipole. While in contractile materials these forces pull the fluid inwards along the axis of the swimmer and push it outwards equatorially, in extensile materials the force distribution is reversed^33^. By systematically varying the strength and nature of activity and the mixture composition, we show here that these two situations lead to qualitatively different instances of exotic active emulsions, with a wide range of surprising and unexpected behaviours. Starting from a lamellar phase (with a 50:50 ratio between the active and passive components), dialling up contractile activity first enhances lamellar ordering, and then creates an emulsion of passive droplets embedded in an active, spontaneously flowing, background. Extensile activity, instead, creates an emulsion of active rotating droplets in a passive matrix. In this case, by varying the mixture composition, we can additionally create “high-internal phase” emulsions, where the active component is dispersed in droplets even when it constitutes the majority phase. All these morphological changes correspond to nonequilibrium phase transitions, whilst the emerging domain size in each phase is mainly controlled by activity.

## Methods

The physics of an exotic polar active emulsion can be described by using an extended version of the well-established active gel theory^1,2,21–23^, in which a set of balance equations governs the evolution of the following hydrodynamic variables: the density of the fluid ***ρ***(**r**, *t*) and its velocity **v**(**r**, *t*), the concentration of the active material ***ϕ***(**r**, *t*) and the polarization **P**(**r**, *t*), which determines its average orientation. (Note that, as in^1^, we assume that the active gel has a constant temperature – i.e., that it is in contact with a reservoir at temperature *T*). The equilibrium properties of the system are encoded in the following Landau-Brazovskii^26,34^ free-energy functional

1 $$\begin{array}{ccc}F[\varphi ,{\bf{P}}] & = & \int {d}^{3}{\boldsymbol{r}}\{\frac{a}{4{\varphi }_{cr}^{4}}{\varphi }^{2}{(\varphi -{\varphi }_{0})}^{2}+\frac{k}{2}{|\nabla \varphi |}^{2}+\frac{c}{2}{({\nabla }^{2}\varphi )}^{2}-\frac{\alpha }{2}\frac{(\varphi -{\varphi }_{cr})}{{\varphi }_{cr}}{|{\bf{P}}|}^{2}\\  &  & +\,\frac{\alpha }{4}{|{\bf{P}}|}^{4}+\frac{\kappa }{2}{(\nabla {\bf{P}})}^{2}+\beta {\bf{P}}\cdot \nabla \varphi \}\end{array}$$

where the first term, multiplied by the phenomenological constant *a* > 0, describes the bulk properties of the lamellar fluid while the second and third terms determine the interfacial tension. Note that here *k* < 0 in order to favour the formation of interfaces while a positive value of *c* guarantees the stability of the free-energy^26^. This describes the properties of a lamellar phase obtained by dispersing a surfactant whose relaxation time is much smaller than that of the other two phases of the mixture (see the Appendix for further details). The bulk term is chosen in order to create two free energy minima, *ϕ* = 0 and *ϕ* = *ϕ*_0_ corresponding respectively to the passive and active phase. Note that *ϕ*_*cr*_ = *ϕ*_0_/2, where *ϕ*_*cr*_ is the critical concentration for the transition from isotropic (|**P**| = 0) to polar (|**P**| > 0) states. The bulk properties of the polar liquid crystal are instead controlled by the |**P**|^2^ and |**P**|^4^ terms, multiplied by the positive constant *α*, whereas the energetic cost due to the elasticity is gauged by the constant *κ* (in the single elastic constant approximation^35^). Finally the last term takes into account the orientation of the polarization at the interface of the fluid. If *β* ≠ 0, **P** preferentially points perpendicularly to the interface (normal anchoring): towards the passive (active) phase if *β* > 0 (*β* < 0).

The dynamic equations governing the physics of the system are

2 $$\rho (\frac{\partial }{\partial t}+{\bf{v}}\cdot \nabla ){\bf{v}}=-\,\nabla P+\nabla \cdot {\underline{\underline{\sigma }}}^{total}.$$

3 $$\frac{\partial \varphi }{\partial t}+\nabla \cdot (\varphi {\bf{v}})=\nabla (M\nabla \frac{\delta F}{\delta \varphi }),$$

4 $$\frac{\partial {\bf{P}}}{\partial t}+({\bf{v}}\cdot \nabla ){\bf{P}}=-\underline{\underline{{\rm{\Omega }}}}\cdot {\bf{P}}+\xi \underline{\underline{D}}\cdot {\bf{P}}-\frac{1}{{\rm{\Gamma }}}\frac{\delta F}{\delta {\bf{P}}},$$

in the limit of incompressible fluid. The first one is the Navier-Stokes equation, in which *P* is the ideal gas pressure and ${\underline{\underline{{\boldsymbol{\sigma }}}}}^{total}$ is the total stress tensor, which is the sum of a passive and an active contribution. The former is, in turn, the sum of three terms, the first of which is the viscous stress ${\sigma }_{\alpha \beta }^{viscous}=\eta ({\partial }_{\alpha }{v}_{\beta }+{\partial }_{\beta }{v}_{\alpha })$ and *η* is the shear viscosity (Greek indexes denote Cartesian components). The second term is the elastic stress and is borrowed from the liquid crystal hydrodynamics. It is written as

5 $${\sigma }_{\alpha \beta }^{elastic}=\frac{1}{2}({P}_{\alpha }{h}_{\beta }-{P}_{\beta }{h}_{\alpha })-\frac{\xi }{2}({P}_{\alpha }{h}_{\beta }+{P}_{\beta }{h}_{\alpha })-\kappa {\partial }_{\alpha }{P}_{\gamma }{\partial }_{\beta }{P}_{\gamma },$$

where **h** = *δF*/*δ***P** is the molecular field^35^ and the parameter *ξ* controls whether the liquid crystal molecules are rod-like shaped (*ξ* > 0) or disk-like shaped (*ξ* < 0). In addition it establishes whether the fluid is flow aligning (|*ξ*| > 1) or flow tumbling (|*ξ*| < 1) under shear. The last term includes an interfacial stress and is given by

6 $${\sigma }_{\alpha \beta }^{binary}=(f-\varphi \frac{\delta F}{\delta \varphi }){\delta }_{\alpha \beta }-k{\partial }_{\alpha }\varphi {\partial }_{\beta }\varphi +c[{\partial }_{\alpha }\varphi {\partial }_{\beta }({\nabla }^{2}\varphi )+{\partial }_{\beta }\varphi {\partial }_{\alpha }({\nabla }^{2}\varphi )]-\beta {P}_{\beta }{\partial }_{\alpha }\varphi ,$$

where *f* is the free energy density. The active contribution is the sole term not stemming from the free energy and is given by^11^

7 $${{\boldsymbol{\sigma }}}_{\alpha \beta }^{active}=-\,{\rm{\zeta }}\varphi ({P}_{\alpha }{P}_{\beta }-\frac{1}{2}|{\bf{P}}{|}^{2}{\delta }_{\alpha \beta }),$$

where *ζ* is the activity strength that is positive for extensile systems and negative for contractile ones. Note that in the derivation of the active stress tensor higher order terms (such as ∂_*α*_*P*_*β*_) are allowed by symmetry. Our assumption is anyway acceptable as the degree of asymmetry of elongated fibres (e.g. actin filaments) is supposed to be small.

Eqs 3–4 govern the evolution of the concentration of the active material and of the polarization field, respectively. The former is a convection-diffusion equation in which *M* is the mobility and *μ* = *δF*/*δϕ* is the chemical potential. The latter is an advection-relaxation equation where Γ is the rotational viscosity, $\underline{\underline{D}}=(\underline{\underline{W}}+{\underline{\underline{W}}}^{T})\mathrm{/2}$ and $\underline{\underline{{\rm{\Omega }}}}=(\underline{\underline{W}}-{\underline{\underline{W}}}^{T}\mathrm{)/2}$ represent the symmetric and the antisymmetric part of the velocity gradient tensor $\underline{\underline{W}}=\nabla {\bf{v}}$. These contributions are in addition to the material derivative as the liquid crystal can be rotated or aligned by the fluid^36^. All other numerical details and the mapping to physical values are given in the Appendix.

## Results

### Contractile mixture

#### Lamellar ordering

**Figure 1:**
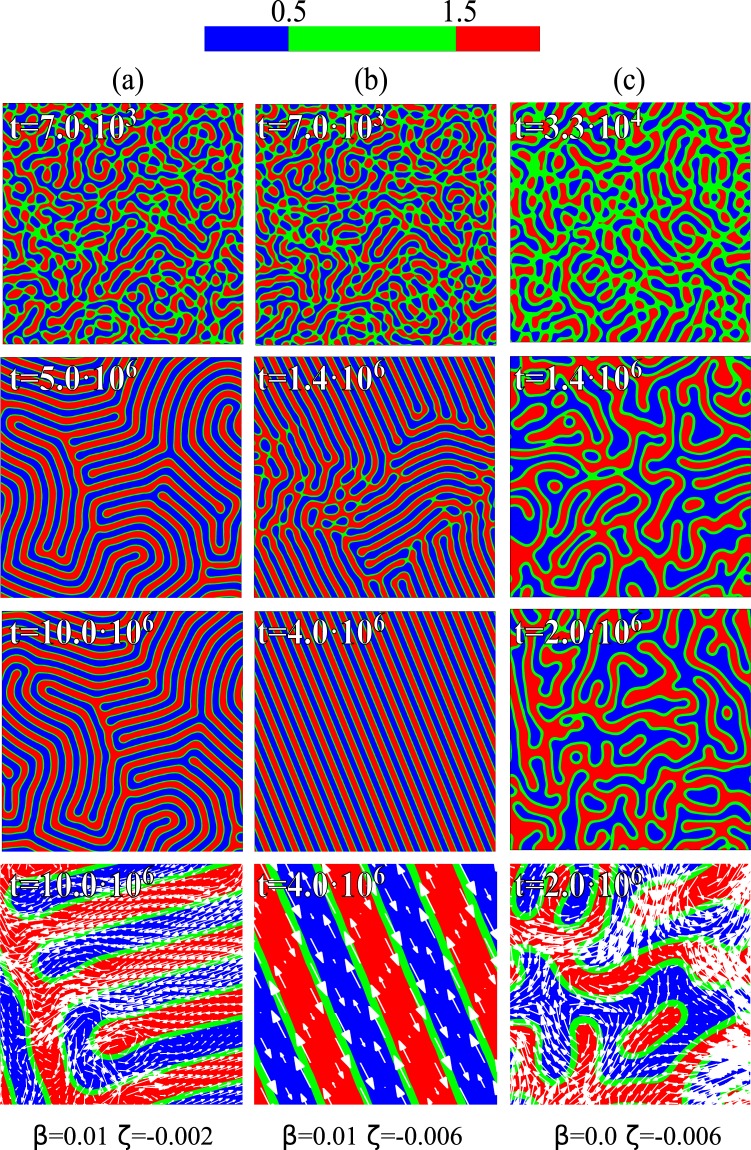
Contour plots of *ϕ* at various times, for 50:50 composition mixtures with small contractile activity. Each column represents a different physical situation: (**a**) and (**b**) show a contractile emulsion with normal anchoring and a different value of *ζ*; (**c**) shows a contractile emulsion with no anchoring. In (**b**) normal anchoring and moderate activity favour the formation of a regular array of lamellae. The last row shows a zoom of the velocity field in the system. These simulations have been performed on a square lattice of size *L* = 128. The color range of the contour plot is the same for all figures and is defined as follows: red (active phase) if *ϕ* > 1.5, green if 0.5 < *ϕ* < 1.5, blue (passive phase) if *ϕ* < 0.5.

To begin with, we consider a symmetric (50:50) emulsion with weak contractile activity (*ζ* < 0). With normal anchoring (Fig. 1a,b, Supplementry Movies 1 and 2), and starting from a uniform phase with small random fluctuations, a disordered lamellar texture rapidly emerges at early times (Fig. 1a,b, top panels). Later on, the pathway followed is greatly affected by the magnitude of |*ζ*|. If this is small, lamellar ordering arrests at late times: here active forces create vortex-like flows localised at grain boundaries (Fig. 1a, bottom panel). For moderate activity the structure orders into a regular, defect-free, array of lamellae with large spontaneous interfacial flow.

**Figure 2:**
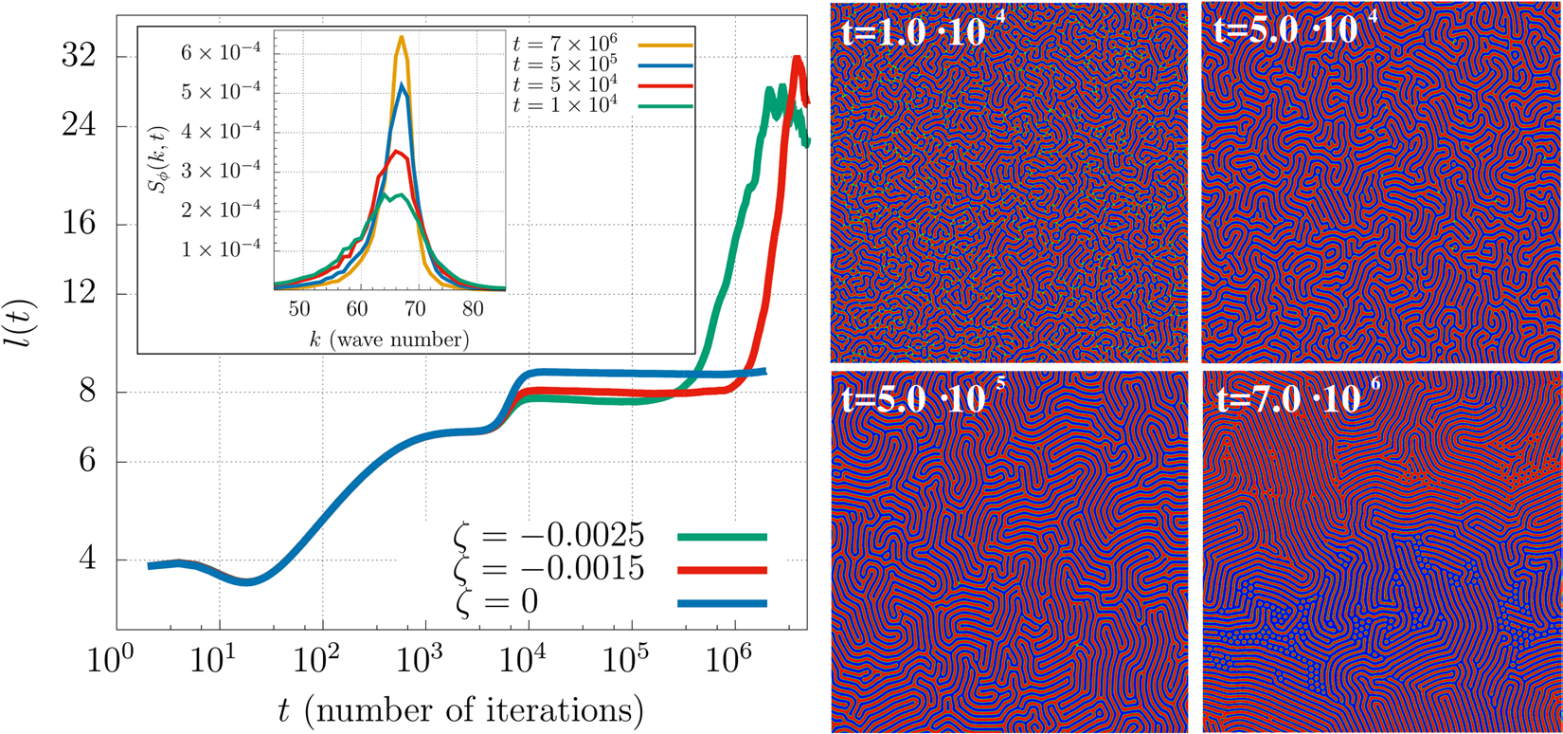
(Left) Time-dependent coarsening length *l*(*t*) for a 50:50 composition, *β* = 0.01 and for *ζ* = 0 (blue), *ζ* = −0.0015 (red) and *ζ* = −0.0025 (green). While in passive systems phase ordering arrests relatively quickly, higher values of contractile activity speed up the dynamics favouring the formation of more ordered stack of parallel lamellae. The inset shows the time evolution of *S*_*ϕ*_(*k*, *t*) for *ζ* = −0.0015. After the initial regime, the position of its peak is found roughly at the same value of the wave vector *k*, corresponding to the lamellar periodicity; the narrowing of its width signals the increase of order in the system. (Right) Time evolution of *ϕ* for *ζ* = −0.0015 and *β* = 0.01. Simulations are performed on a square lattice of size *L* = 512 and wavevector *k* in the inset is labeled in lattice units.

Activity enhances lamellar ordering also for larger system size, although some defects survive until late times. By following a standard approach^37^, the ordering dynamics can be evaluated by looking at the evolution of the typical domain size *l*(*t*). This is estimated by computing the inverse of the first moment of the spherically averaged structure factor *S*_*ϕ*_(*k*, *t*) = 〈*ϕ*(**k**, *t*)*ϕ*(−**k**, *t*)〉_*k*_^37^,

8 $$l(t)=2\pi \frac{\int {S}_{\varphi }(k,t)dk}{\int k{S}_{\varphi }(k,t)dk},$$

where *k* is the modulus of wave vector **k**, 〈⋅〉_*k*_ is an average over a shell in **k** space at fixed *k*, and *ϕ*(**k**, *t*) is the spatial Fourier transform of *ϕ*(**r**, *t*). Figure 2 (left) shows *l*(*t*) and the time evolution of *S*_*ϕ*_ of a symmetric mixture for different values of small contractile activity. Regardless of the value of *ζ*, the crossover from the early-time diffusion regime to the late one is always sigmoidal, and lasts up to $t\simeq {10}^{4}$ where a short plateau is observed. Later on ordering restarts, but while for *ζ* = 0 *l*(*t*) quickly saturates to a constant value, for higher values of *ζ*, after a temporary saturation (the second plateau from $t\simeq {10}^{4}$ to $t\simeq 3\times {10}^{5}$), it rapidly grows as the velocity field becomes strong enough to disentangle the intertwined domain pattern. However, due to the high number of topological defects in large systems, a full defect-free array of lamellae is difficult to achieve. At very late times *l*(*t*) may saturate even for the active material, with the formation of few active droplets coexisting with lamellae in a passive polar background. A further investigation of the structure factors while varying *ζ* makes clear that contractile activity also entails lamellar width, leading to progressively thinner lamellae as it is raised (Section 1 of SI (See Supplemental Material for additional results and movies)).

Lamellar ordering is also affected by the strength of the anchoring. If this is absent, lamellae get disrupted, and domains either partially coarsen, or break up into droplets (Fig. 1c, Supplementry Movie 3). At steady state, the mixture is morphologically closer to a bicontinuous phase, reminiscent of arrested spinodal decomposition. The active flow pattern is significantly different from those in Fig. 1a,b: rather than being confined at the boundary between lamellae, the flow is now driven by deformations in the polarisation which tend to occur inside active domains.

#### Contractile emulsion for symmetric composition at high activity

**Figure 3:**
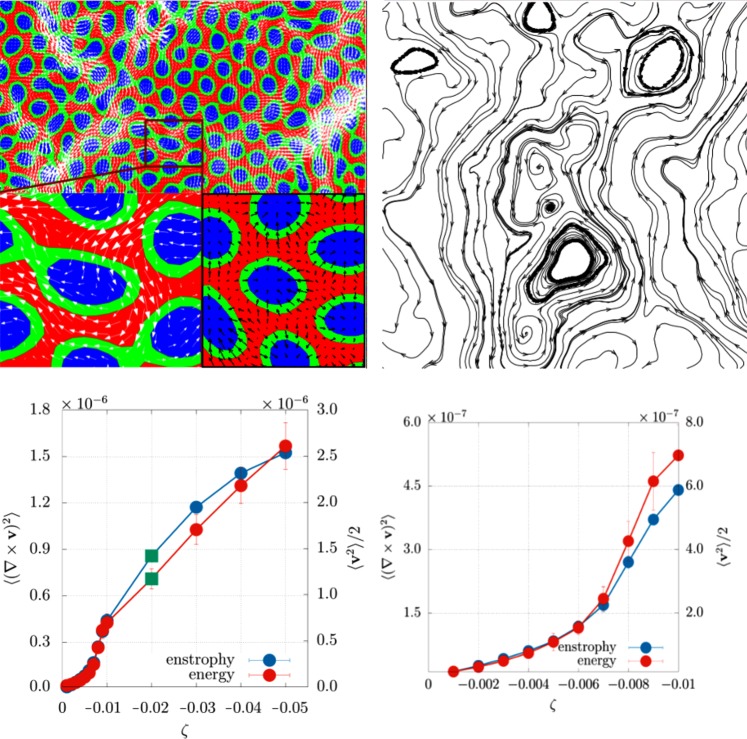
(Top, left) Superposition of the velocity field (white arrows) and of the polarisation field (black arrows) to the contour plot of *ϕ* in a portion of a 50:50 mixture with strong contractile activity (*β* = 0.01, *ζ* = −0.02) at late times, showing a self-assembled emulsion of passive droplets in an active matrix. (Top, right) Zoom with streamlines of the flow field. (Bottom, left) Plot of the mean kinetic energy and of the mean enstrophy versus *ζ*. (Bottom, right) Zoom of both plots for small values of contractile activity. Simulations were performed on a square lattice of size *L* = 256.

Systematic scanning of the activity for fixed normal anchoring (Fig. 3 and S1 (See Supplemental Material for additional results and movies)) reveals the existence of another morphology for stronger activity, a self-assembled emulsion of passive droplets in an active matrix. The spontaneous flow within the active background keeps stirring the system (Fig. 3, top panels, for typical flow field an polarisation plots), so that the passive droplets never settle into a static pattern. The flow profile is compatible with active turbulence^38,39^. The transition between lamellar and droplet emulsions occurs at *ζ* ∼− 0.007, a point of a sharp change in the behavior of the mean enstrophy (defined as the mean squared vorticity 〈(∇ × **v**)^2^〉, where 〈⋅〉 denotes the average over the system) and of the kinetic energy (see Fig. 3, bottom).

An analysis of the evolution of the morphology with time (Supplementry Movie 4 (See Supplemental Material for additional results and movies)), or of the steady state pattern with activity (Fig. S1 (See Supplemental Material for additional results and movies)), suggests a mechanism for the transition between lamellar and droplet emulsion. If the activity is strong enough, undulations at the lamellar boundary favour interfacial splay, which in turn leads to active forces pinching off lamellae into droplets. [The dimensionless parameter controlling the pinch-off instability should therefore be *ζl*/*σ*, with *l* the Brazovskii domain size and *σ* the effective surface tension.] But why do passive, rather than active, droplets form? The reason is that splay creates inward asters for the polarization field: placing an isotropic droplet in the middle therefore relieves elastic stresses (Fig. 3, bottom right). The mechanism is therefore to some extent similar to the one which generically drives nanoparticles towards disclinations and defects in liquid crystals^40,41^. Inspection of the structure factor of the droplet emulsions found at large |*ζ*| additionally suggests that the steady-state domain size decreases with contractile activity (Fig. S2 (See Supplemental Material for additional results and movies)).

### Extensile mixture

#### Extensile emulsion for symmetric composition

**Figure 4:**
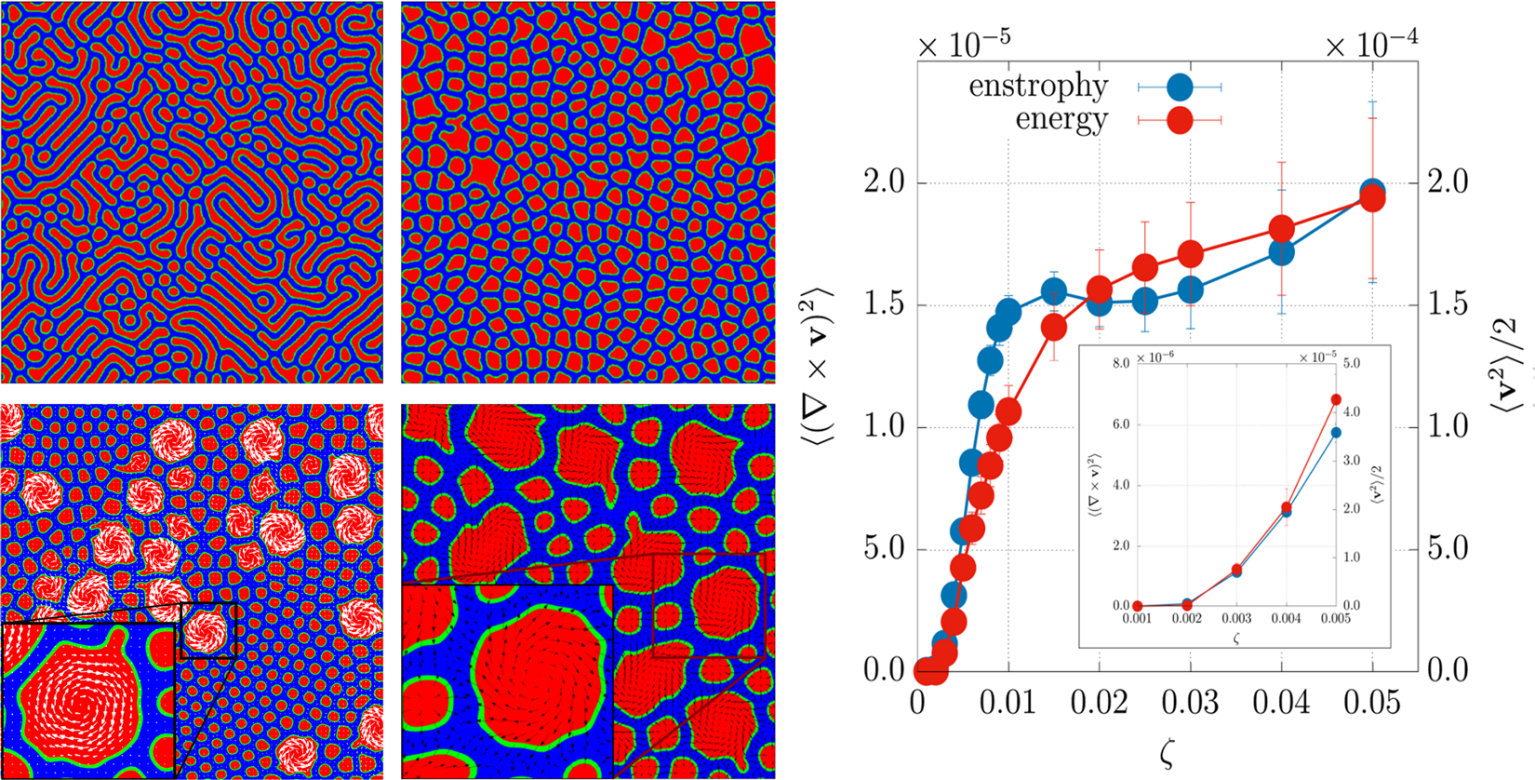
(Left) Late time contour plots of *ϕ* for 50:50 extensile mixtures at *β* = 0.01. (Top, left) *ζ* = 0.001; (top, right) *ζ* = 0.002; (bottom, left) *ζ* = 0.003; (bottom right) *ζ* = 0.004. Extensile activity leads to an emulsion of active droplets within a passive background. For moderate activity larger droplets rotate, as depicted by white velocity vectors in the bottom left panel. The zoom at *ζ* = 0.004 illustrates typical polarisation patterns (black vectors in bottom the right panel) that have either an aster-like structure in small non-rotating droplets or a spiral shape in big rotating ones. (Right) Plot of the mean kinetic energy and of the mean enstrophy for several values of the extensile activity. The inset shows the behavior of both plots for small values of *ζ*. Simulations were performed on a square lattice of size *L* = 256.

We now consider the case in which the active component is extensile. Switching the sign of the activity parameter creates completely different patterns. Upon increasing *ζ* the lamellar phase gives way to an emulsion of active droplets within a passive background (Figs 4 and S4 (See Supplemental Material for additional results and movies)) – the inverse of what happens in contractile mixtures. The active droplet emulsion is approximately monodisperse for *ζ* = 0.002 (Fig. 4, top right), and bidisperse for *ζ* = 0.003 (Fig. 4, bottom left). The transition between the lamellar and emulsion morphologies corresponds to the value of activity for which the mean enstrophy and kinetic energy depart from zero, ${\zeta }_{c}\sim 0.002$ (Fig. 4). Analysis of the mixture structure factor further shows that the droplet size increases monotonically with *ζ* (see Section 2 of the Supplementary Information) – again the opposite behaviour of contractile mixtures. At very large *ζ* (as long as $\zeta \mathop{ < }\limits_{ \tilde {}}0.02$) there is a crossover to macroscopic phase separation between active and passive components (corresponding to the plateau in the enstrophy and kinetic energy plots in Fig. 4), whereas for higher values of *ζ* the demixing process stops.

#### Polarisation pattern and angular velocity in extensile emulsion

**Figure 5:**
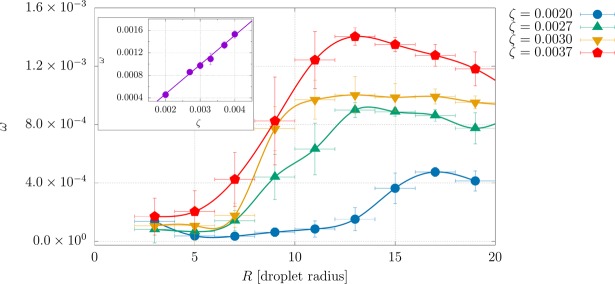
Angular velocity *ω* calculated as a function of the radius *R* of the droplet for four different values of the activity strength (*ζ* = 0.002 blue/circles, *ζ* = 0.0027 green/triangles, *ζ* = 0.003 yellow/reverse triangles and *ζ* = 0.037 red/pentagons). For all cases the anchoring strength is fixed to *β* = 0.01. Each value *ω* is calculated as an average value over a class of droplets (of at least six uncorrelated configurations at late times) whose radius ranges from *R* − 1 to *R* + 1 around a defined radius *R*. This also defines the error bar on *x*-axis. The error bar on the *y*-axis is the standard deviation of the average. Note that, at fixed *ζ*, the angular velocity increases with *R* up to $R\simeq 13$ for *ζ* = 0.0027, 0.003, 0.0037, and up to $R\simeq 15$ for *ζ* = 0.002. Then it remains approximately constant. In this region the mean angular velocity grows linearly with *ζ* (see the inset).

Unlike the case of contractile mixture, the active component is now within the droplets because extensile activity and normal anchoring lead to a purely rotational flow in the droplet itself, creating rotating spirals similar to those described in^42–45^. Without this sustained flow, the internal spiral defect would be elastically unstable, as was the case for the contractile mixture (and this explains the inversion of the emulsion). In the bottom right panel of Fig. 4 we show the typical polarisation patterns observed inside an extensile droplet for *β* = 0.01, *ζ* = 0.004. Polarisation in smaller (non-rotating) droplets arranges into an aster-like pattern due to frustration caused by the strong perpendicular anchoring, whereas in larger droplets the high activity yields to the formation of strong bend distortions (the typical elastic instability in extensile materials^1^), which competes with the surface anchoring. These distortions give rise to spirals in the polarization field and to a vortex-like fluid flow pattern (as that reported in Fig. 4) which triggers a spontaneous rotation of extensile droplets when the ratio *ζR*^2^/*κ* exceeds a critical threshold, that we find approximately equal to 13.8 (±10%). This is consistent with values found in previous works^43–46^, where *ζR*^2^/*κ* is estimated to range within 1–10^4^ and to strongly depend on the size *R*. A natural question to address is how the angular velocity *ω* of these droplets depends upon their radius *R* (the distance from the centre of mass of the droplet) and upon the activity strength *ζ*. In Fig. 5 we report a late time estimate of *ω* as a function of *R* for five different values of *ζ*. We calculate the angular velocity as $\omega =\frac{\int dV(\varphi /{R}^{2}){\bf{r}}\times {\bf{v}}}{\int dV\varphi }$, averaged over a class of droplets whose radius *R* has a bin width of two lattice sites around it. Clearly *ω* increases both with *ζ* (as the system possesses higher kinetic energy) and with *R*, up to $R\simeq 13$ for *ζ* = 0.0027, 0.003, 0.0037, whereas afterwards it remains roughly constant. In this region dimensional analysis suggests that $\omega \sim \zeta /\eta$ (with *η* effective viscosity); this is also shown in the inset of Fig. 5, where the mean angular velocity has been plotted versus *ζ*. At higher activities the flow field considerably affects the shape of the droplets, many of which fluctuate greatly over time, and either merge into larger domains split up (see Supplementry Movies 5, 6 (See Supplemental Material for additional results and movies)).

#### Extensile emulsion for asymmetric composition and phase inversion

**Figure 6:**
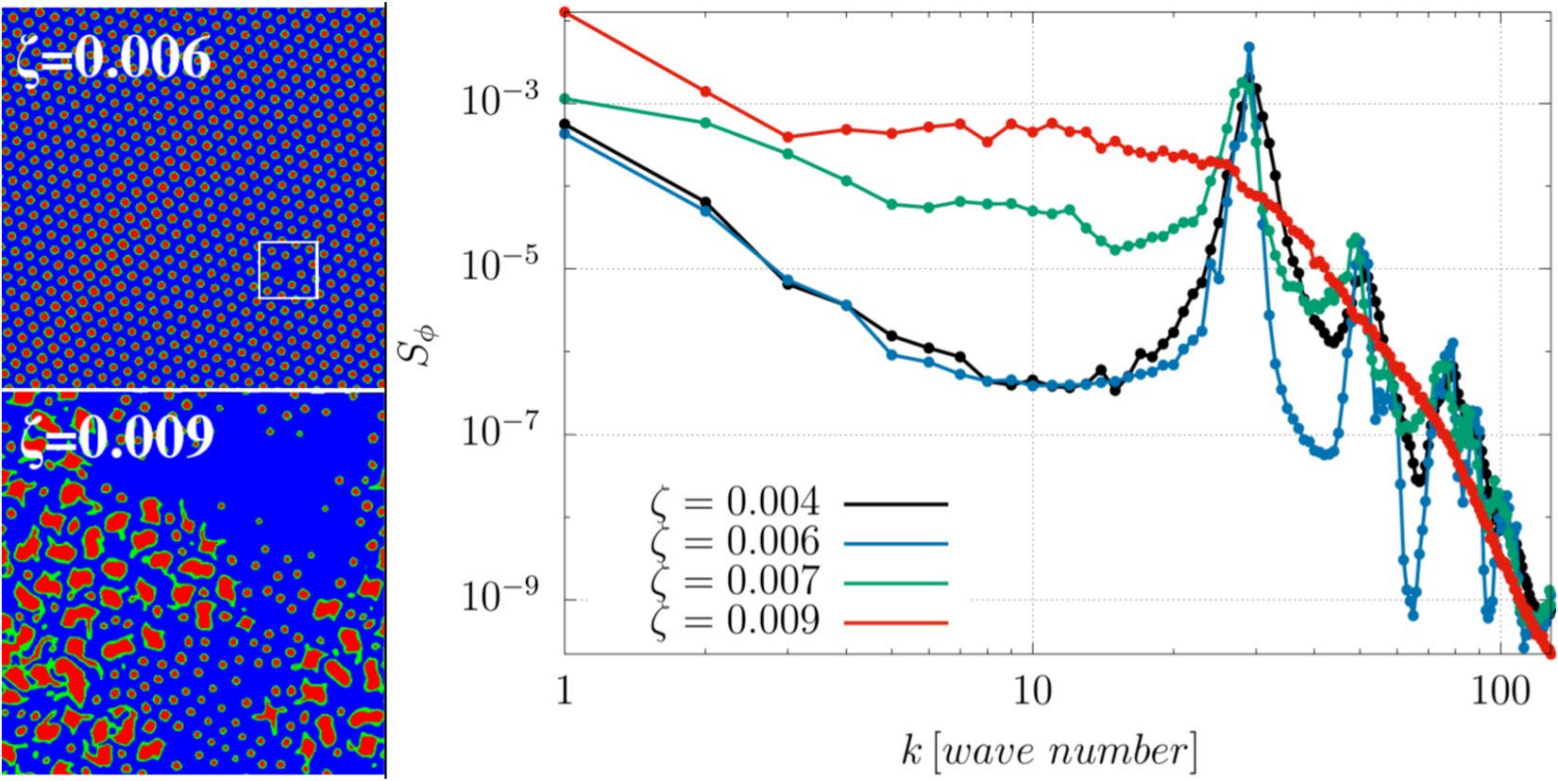
(Left) Snapshots of *ϕ* contour plots with 10:90 composition, at late times for different values of *ζ* in an extensile material with *β* = 0.01. An emulsion of active droplets forms in a passive matrix for all values of *ζ* considered. While at low activity ($\zeta \mathop{ < }\limits_{ \tilde {}}0.005$) droplets arrange in an almost rigid lattice structure with defects, a more severe active doping is capable to maintain a residual flow that removes defects from the system (top left panel). If activity exceeds a critical threshold (${\zeta }_{c}\simeq 0.007$), larger dynamical domains form from collisions and merging of smaller droplets (left bottom panel). (Right) This panel shows the concentration structure factor for different values of *ζ*: peaks observed at *k* = 30, *k* = 50 and *k* = 80 correspond to *l* = 8.5, *l* = 5.1 and *l* = 3.2, respectively. The first one gauges the triangular lattice size while the others are two different characteristic lengths of the domains observed. Wavevector axis is labeled in lattice units. Results refer to a square lattice of size *L* = 256.

All results so far pertain to systems with a 50:50 ratio between active and passive component. By changing the composition yields similar phenomenology. Focussing for concreteness on extensile mixtures, a 10:90 emulsion (where the first number refers to the active component) shows transitions from a lattice of non-motile droplets (the equilibrium configuration for a passive asymmetric mixture) to an active rotating droplet emulsion, with droplet size increasing with *ζ* as for 50:50 extensile mixtures (left panels in Fig. 6). Droplets arrange in an hexatic order with some defects, as long as $\zeta \simeq 0.005$; if the activity augments up to 0.006, a defect-free configuration emerges, except for a vacancy in the droplet lattice (highlighted by a white square in the upper left-hand panel of Fig. 6). For more intense active doping ($\zeta \mathop{ > }\limits_{ \tilde {}}0.007$) the hexatic order is lost and large domains (of radius $R\simeq 10$) of extensile material start to form from the merging of several droplets. Like the symmetric case they acquire spontaneous rotation, a motion sustained by the combination of strong interface anchoring and intense bend distortions of the polarisation field. When the activity is very intense (*ζ* = 0.009), a higher number of large dynamic domains is created (see Fig. 6 right panel), while the smaller ones progressively shrink by merging/collision and probably via Ostwald ripening^47^.

**Figure 7:**
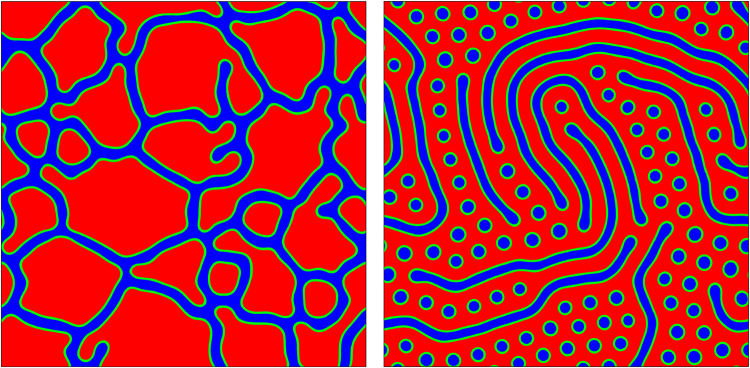
Contour plots of *ϕ* for an 80:20 emulsion and *β* = 0.01. (Left) Late time configuration for *ζ* = 0.0015. (Right) Configuration resulting from dynamic phase inversion. This is obtained starting from the configuration on the left, then imposing a larger activity (*ζ* = 0.008), letting the system evolve (for 10^5^ timesteps), and finally switching the activity off (*ζ* = 0). Simulations have been performed on a square lattice of size *L* = 128.

Increasing the active component fraction beyond 50% leads to additional interesting dynamics, as it creates high internal phase emulsions, i.e. systems where the majority (active) phase forms droplets (Fig. 7, for a 80:20 emulsion, and Fig. S4 (See Supplemental Material for additional results and movies) for a 70:30 emulsion). If the activity is first increased and then switched off, the emulsion undergoes a dynamic phase inversion, to leave a suspension of isotropic islands and droplets, within a liquid crystalline sea (see Fig. 7). The pathway to phase inversion involves first rapid droplet coalescence, due to the turbulent spontaneous flow which arises at high activity (See Supplemental Material for additional results and movies), followed by a slow reorganization driven by the chemical potential and thermodynamic stress tensor, when activity is switched off. This phenomenon is reminiscent of phase inversion in emulsions^48^, although in our case this is triggered by variation of a nonequilibrium parameter (the activity).

## Conclusions

In summary, here we have studied the behaviour of an emulsion obtained by mixing an isotropic fluid with an active polar gel, in the presence of a surfactant. The thermodynamic free energy density favours normal anchoring at the interface, as well as the formation of a lamellar phase, for symmetric passive mixtures. Our simulations predict that the resulting composite materials should display a striking variety of exotic phase behaviours and morphologies: remarkably, any one of these can be selected by tuning *activity alone*. Thus, we find that a moderate contractile activity (e.g., corresponding to emulsions containing actomyosin as the active ingredient) sets up interfacial shear flows which enhance and speed up lamellar ordering. Increasing the strength of contractile activity disrupts the passive lamellar ordering to create emulsions of passive droplets within an active self-stirring background. Instead, we predict that extensile activity (for instance corresponding to mixtures where the active component is a bacterial fluid) should lead to the self-assembly of a polydisperse suspension of active rotating droplets in a passive background. Such a phase may be relevant to the understanding of the self-assembled bacterial rotors reported in^27^. By tuning the overall mixture composition, we can also stabilize emulsions in which the majority active phase is dispersed as droplets. This state rapidly undergoes phase inversion as soon as activity is switched off.

Active emulsions like those described here may be self-assembled, by varying component composition, in water-oil emulsions containing actomyosin, or extensile microtubule-motor suspensions^5^. In the current formulation, the latter active mixture always adsorbs onto the water-oil interface, rather than dispersing inside the aqueous phase^5^, and the long term stability is also a potential practical problem. However it might be possible to overcome such technical issues in the future – for instance, hydrodynamic coupling to another structured fluid as in^6,49^ might be exploited to create active interfacial droplets at the 2D interface. In that case, the surprising richness of behaviour of our exotic active emulsions could be exploited to design active shape-changing microfluidic systems, or to self-assemble soft composite materials with tunable morphology.

## Appendix

### Numerical details and mapping to physical values

Eqs 2–4 are numerically solved by using a hybrid lattice Boltzmann method (in the limit of incompressible flow), successfully tested in previously studied systems such as binary fluids^50^, liquid crystals^51,52^ and active fluids^21–23,53,54^. It consists in solving the Navier-Stokes equation via a standard lattice Boltzmann approach while Eq. 3 and Eq. 4 are integrated via a finite-difference predictor-corrector algorithm. Simulations are performed on a two-dimensional square lattice whose linear size ranges from *L* = 128 to *L* = 512. The system is initialized in a mixed state, with *ϕ* uniformly distributed between 1.1 and 0.9 (in symmetric mixtures). The concentration *ϕ* ranges from *ϕ* = 0 (passive phase) to $\varphi \simeq 2$ (active phase), that correspond to the two minima of the double-well potential. The starting polarization field **P** is randomly distributed between 0 (passive phase) and 1 (active phase). Unless otherwise stated, parameter values are *a* = 4 × 10^−3^, *k* = −6 × 10^−3^, *c* = 10^−2^, *α* = 10^−3^, *κ* = 10^−2^, Γ = 1, *ξ* = 1.1, *ϕ*_0_ = 2, *η* = 1.67. Within our model, the surfactant is modeled implicitly and it is therefore non-trivial to estimate its exact required volume fraction to recreate the systems we simulate in the lab. Inspection of experimental phase diagrams for passive oil-water emulsions suggests that, for instance, symmetric emulsions at room temperature may be obtained with a ∼20–30% volume fraction of a suitable surfactant^55^ (though the exact value is very sensitive to the chemical nature of the surfactant employed and to the temperature.) By following previous studies^21,22,54^, an approximate relation between simulation units and physical ones (such as those of a contractile active gel) can be obtained by using as length-scale, time-scale and force-scale respectively the values *L* = 1 *μ*m, *τ* = 10 ms and *F* = 1000 nN (see Table 1). Note that, as in previous Lattice Boltzmann simulations, the fluid mass density *ρ* is much larger than the mass density of a real solvent (such as water)^56^. This assumption that is valid as long as inertial effects are negligible compared to viscous ones, reduces the computation time by several orders of magnitude. Throughout our simulations the Reynolds number, for the case in which the droplets are observed, is evaluated in terms of the average droplet radius, of the viscosity and of the velocity of the fluid. It remains below 0.1, a value in which inertial effects are indeed negligible.

**Table 1:** Typical values of the physical quantities used in the simulations.

Model variables and parameters	Simulation units	Physical units
Effective shear viscosity, *η*	5/3	1.67 kPas
Effective elastic constant, *κ*	0.006	6 nN
Shape factor, *ξ*	1.1	dimensionless
Effective diffusion constant, *D* = *Ma*	0.0004	0.004 *μ*m^2^ s^−1^
Rotational viscosity, Γ	1	10 kPas
Activity, *ζ*	0−0.01	(0–100) kPa

## Supplementary information

Supplementary Information

Movie 1

Movie 2

Movie 3

Movie 4

Movie 5

Movie 6
