# Supplementary material for: Lamellar ordering, droplet formation and phase inversion in exotic active emulsions
Source: Sci Rep. 2019 Feb 26;9:2801. doi: 10.1038/s41598-019-39190-6 (PMC6391428; doi:10.1038/s41598-019-39190-6)
Supplement: Supplementary file 1 — Supplementary Information [file 41598_2019_39190_MOESM1_ESM.pdf]

Lamellar ordering, droplet formation and phase inversion in exotic active emulsions. Supplementary Information.

F. Bonelli[1], L. N. Carenza[2], G. Gonnella[2], D. Marenduzzo[3], E. Orlandini[4], A. Tiribocchi[5]

[1] Dipartimento di Meccanica, Matematica e Management, DMMM, Politecnico di Bari, 70125 Bari, Italy
[2] Dipartimento di Fisica and Sezione INFN, Università di Bari, Via Amendola 173, 70126 Bari, Italy
[3] SUPA, School of Physics and Astronomy, University of Edinburgh, Edinburgh EH9 3JZ, United Kingdom
[4] Dipartimento di Fisica e Astronomia, Università di Padova, 35131 Padova, Italy
[5] Center for Life Nano Science @Sapienza, Istituto Italiano di Tecnologia, Viale Regina Elena, 295, I-00161 Roma, Italy

1. Morphology of contractile emulsions for $50:50$ composition

As discussed in the main text, if the activity is sufficiently high, an emulsion of passive droplets assembles in an active contractile background. In order to demonstrate how the lamellar-to-droplet transition occurs, we show late-time configurations of ϕ for $50:50$ composition and for different values of ζ (Fig. 1). While for low values of activity the typical intertwined

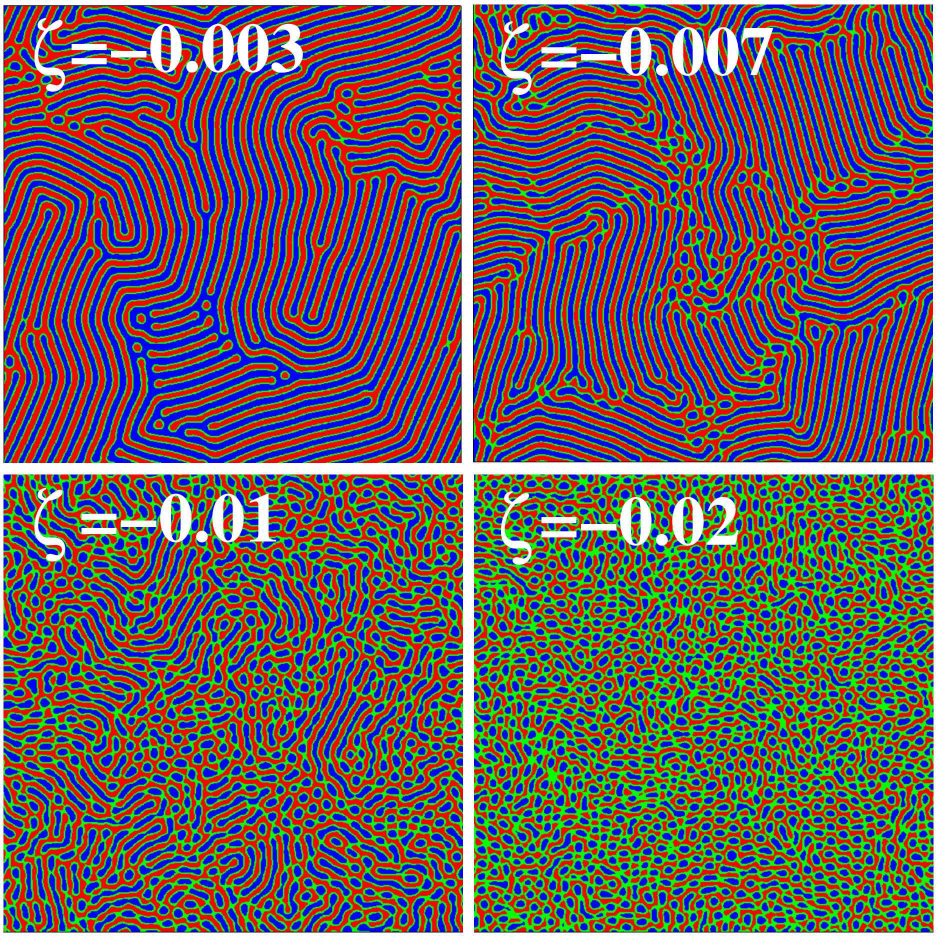

Fig.S 1. Transition from lamellar to emulsion phase at increasing contractile activity. We show four snapshots of ϕ contour plots for $50:50$ composition at late times and for different values of ζ in a contractile material with $\beta = 0.01$. While for relatively small values of the activity ($\zeta = -0.003, -0.007$) lamellar ordering dominates, an emulsion of passive material embedded in the active background emerges for higher values ($\zeta = -0.01, -0.02$). Results refer to a square lattice of size $L = 256$.

lamellar pattern dominates (Fig. 1, $\zeta = -0.003$), for increasing values of ζ small isles of passive material emerge (Fig. 1, $\zeta = -0.007$).Further increasing ζ, the mixture undergoes a non-equilibrium transition to an emulsion phase, in which a dynamic array of passive droplets is created in an active matrix (Fig. 1, $\zeta = -0.01, -0.02$ and supplementary movie 4). A

quantitative estimate of the evolution of the characteristic lengthscale l observed by varying ζ can be obtained by looking at the concentration structure factor $S_\phi(k,t)$. It shows a peak at $k \simeq 32$ for $\zeta = -0.001$, which shifts towards higher values (e.g. $k \simeq 35$ and $k \simeq 38$) for increasing activity (Fig. 2a). They correspond to a lengthscale $l = L/k \simeq 8$, 7 and 6.7 respectively, an estimate of the characteristic size either of the lamellae (for low values of ζ) or of the droplets in the emulsion phase (at high ζ). Note that the steady-state size of the droplets diminishes with increasing contractility. A further peak appears at $k \simeq 100$ corresponding to $l \simeq 2.5$, an estimate of the size of the lamellar interface observed for low values of ζ. For higher values the emulsion phase dominates and only one characteristic lengthscale appears. The velocity field structure factor $S_v(k,t)$ (calculated as $S_v(k,t) = \langle \mathbf{v}(\mathbf{k},t)\mathbf{v}(-\mathbf{k},t)\rangle_k$), besides the two peaks at $k \simeq 35$ and $k \simeq 100$, displays two further ones at $k \simeq 80$ and $k \simeq 105$ at $\zeta = -0.001$ and $\zeta = -0.005$, corresponding to $l \simeq 3.2$ and $l \simeq 2.4$. These indicate the characteristic sizes where intense fluid flows, directed along opposite directions and mainly located at the lamellar interface, are expected to form. These peaks appear only for small and intermediate values of ζ whereas they are progressively washed off for high values of ζ.

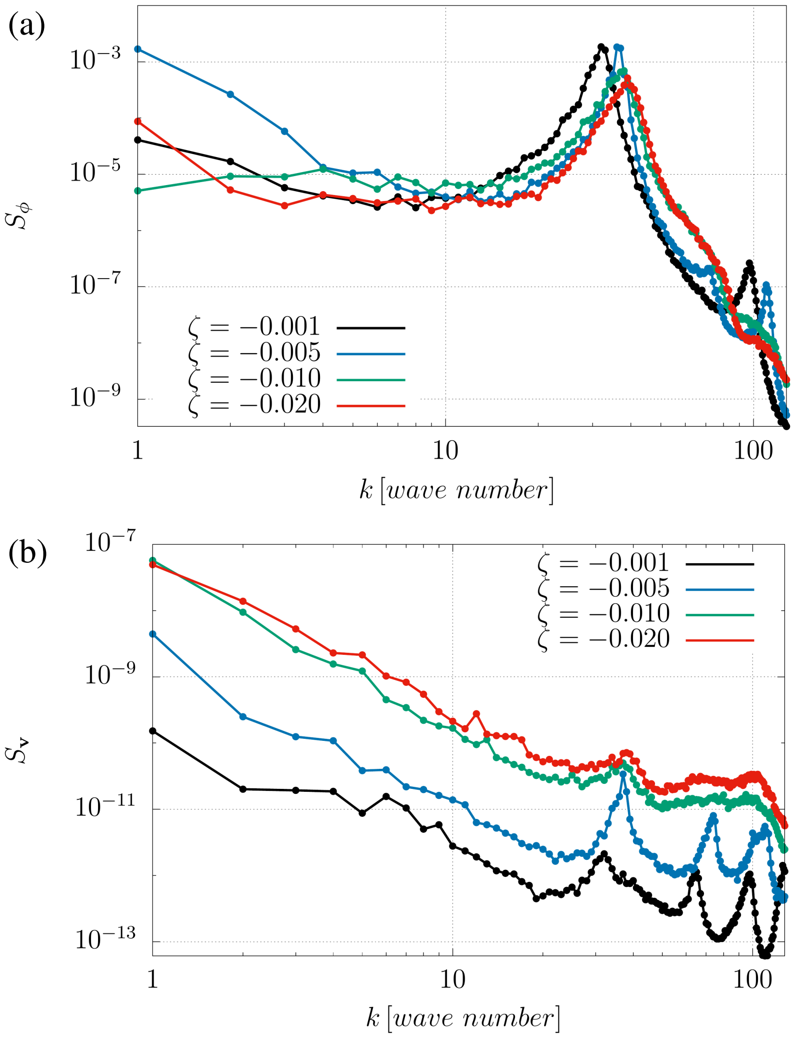

Fig.S 2. Concentration and velocity structure factor of symmetric contractile mixtures. (a) Concentration structure factor for different values of ζ in a contractile material. An approximately stable peak at $k \simeq 35$ (corresponding to $l = L/k \simeq 7$) is observed for all ζ considered. A further peak appears at $k \simeq 100$, corresponding to $l \simeq 2.5$, only for $\zeta = -0.001$ and $\zeta = -0.005$. (b) Velocity structure factor for the same values of ζ. Four peaks appear for $\zeta = -0.001$ and $\zeta = -0.005$ at $k \simeq 38$ ($l \simeq 7$), $k \simeq 80$ ($l \simeq 3.2$), $k \simeq 100$ ($l \simeq 2.5$) and $k \simeq 105$ ($l \simeq 2.4$), which disappear for higher values of the activity. Results refer to a square lattice of size $L = 256$. Wavevector axis is labeled in lattice units.

2. Morphology of extensile emulsions for $50:50$ composition

Unlike the case of contractile materials, if the active component is extensile the lamellar phase is lost, and even for small values of ζ and starting from an initially uniform mixture, an emulsion of active droplets assembles in the passive fluid. In Fig. 3 we show the transition from a structure in which pieces of lamellae can be still observed at $\zeta = 0.001$ towards an active emulsion phase in which rounded-shaped domains acquire rotational speed with increasing activity. The calculation of the concentration

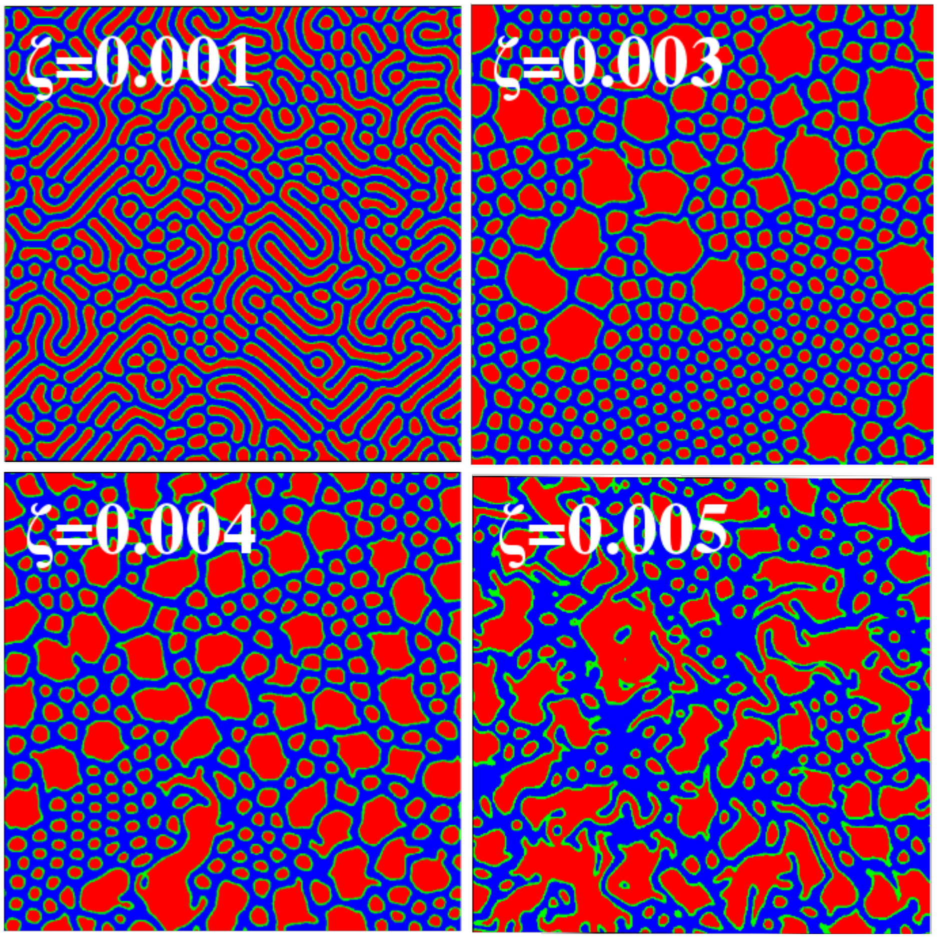

Fig.S 3. Transition from lamellar to emulsion phase at increasing extensile activity. Snapshots of ϕ contour plots for $50:50$ composition at late times and for different values of ζ in an extensile material with surface anchoring parameter $\beta = 0.01$. Here small droplets of extensile material form already at low activity ($\zeta = 0.001$). For higher values a bidisperse emulsion emerges ($\zeta = 0.003$), made up by rotating domains whose mean size slightly increases with the activity ($\zeta = 0.004$, $\zeta = 0.005$) due to collisions and coalescence between them. Results refer to a square lattice of size $L = 256$.

structure factor (not shown here) unveils, for instance, a broad peak at $k \simeq 20$ for $\zeta = 0.003$, corresponding to $l \simeq 13$, the characteristic lengthscale of the droplets for this case. The velocity structure factor is approximately a smooth curve in the emulsion phase (i.e. for values of ζ higher than 0.002), as the fluid velocity inside the droplets and along their interface is overall unidirectional (see Fig.3 of the main text). It also shows a peak for $\zeta = 0.001$ at $k \simeq 90$ corresponding to $l \simeq 2.85$, the interfacial lamellar distance. The stronger the activity is the higher the curves are: a further evidence of the increase in the fluid flow strength. Interestingly each curve exhibits a monotonic decreasing trend, suggesting the typical length-scale over which the velocity field develops: in particular for higher values of activity length-scales are comparable to the overall size of the lattice; on the contrary the peaks at large wave-numbers for $\zeta = 0.001$ demonstrate that, in this case, the dynamical response of the fluid develops over the typical droplet length-scales.

3. Phase inversion

In the main text we have shown that if the area fraction of the active gel is 80:20 (80% of active material and 20% of passive material), emulsions rich of droplets in active material are observed. If the activity is switched off, the emulsion undergoes a

phase inversion, a phenomenon in which the dispersed phase becomes the continuous one and vice-versa. Here we corroborate this finding by presenting more results obtained by varying the composition above the critical value 0.5 (see Fig.4) for a different value of ζ. Isolated regions of active material are again observed in a passive background. In particular late times configurations of Fig.4a and c have been obtained for mixtures of respectively 70:30 and 75:25 compositions, both for $\zeta = 0.002$ and $\beta = 0.01$. Although the two cases look quite similar, if then ζ is switched to zero the resulting dynamics is different. In particular, if the composition is 70:30, the late time morphology is a lamellar pattern decorated by small isolated isotropic droplets (see Fig.4b). If, on the other hand, the composition is 75:25, the late time morphology looks more like a phase inversion of isolated islands and droplets dispersed in a continuous polar phase. In this last case β has been set equal to zero (namely without surface anchoring).

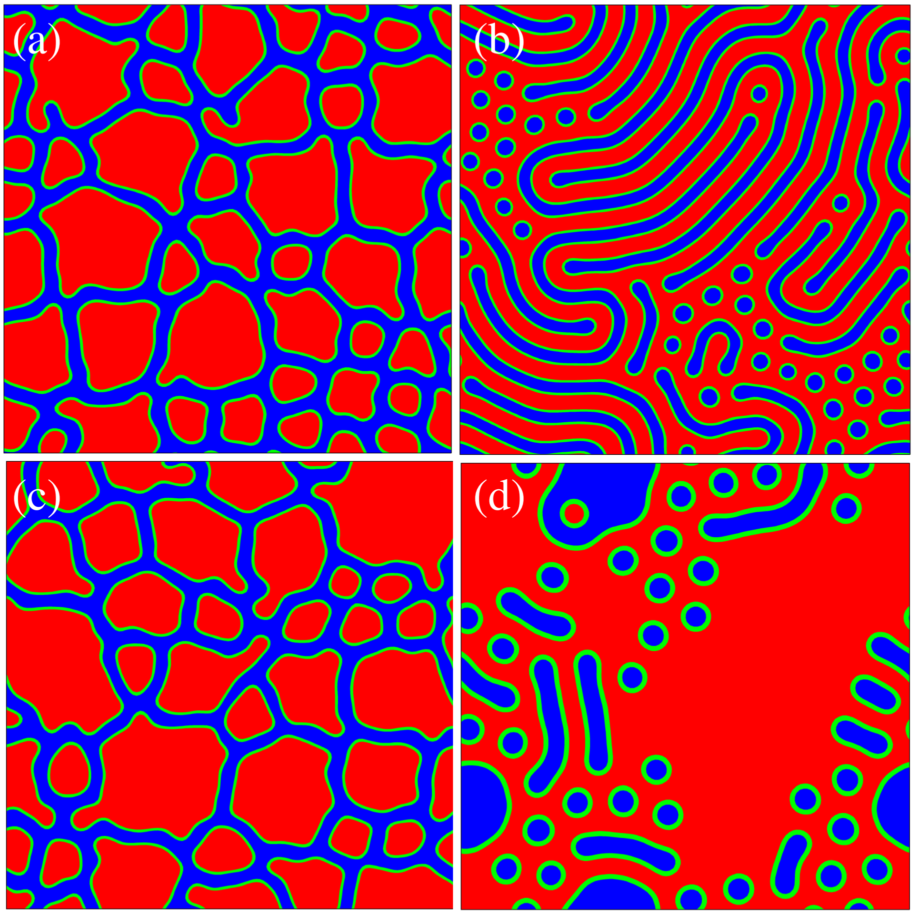

Fig.S 4. Phase inversion in extensile mixtures. Panels (a) and (b) show the plot of the concentration ϕ at times $t = 2 \times 10^6$ and $t = 3.4 \times 10^7$ respectively, for a 70:30 emulsion and $\beta = 0.01$. In (a) $\zeta = 0.002$, whereas in (b), ζ has been switched off to zero. A bicontinuous pattern with isolated isotropic droplets form during time. Panels (c) and (d) show the plot of the concentration ϕ at times $t = 2 \times 10^6$ and $t = 2.2 \times 10^7$ respectively, for a 75:25 emulsion. In (c) $\zeta = 0.002$ and $\beta = 0.01$ whereas in (d) both β and ζ have been switched off to zero. Importantly, here the phase inversion occurs even when in the absence of surface anchoring. The switching off dynamics follows the protocol described in the caption of Fig.4 of the main text. Results refer to a square lattice of size $L = 128$.
